# Defining bacterial species in the genomic era: insights from the genus *Acinetobacter*

**DOI:** 10.1186/1471-2180-12-302

**Published:** 2012-12-23

**Authors:** Jacqueline Z-M Chan, Mihail R Halachev, Nicholas J Loman, Chrystala Constantinidou, Mark J Pallen

**Affiliations:** 1 Institute of Microbiology and Infection, School of Biosciences, University of Birmingham, Edgbaston, B15 2TT, UK

**Keywords:** Genome-based taxonomy, Bacteria, Sequence-based analysis, Whole-genome data

## Abstract

**Background:**

Microbial taxonomy remains a conservative discipline, relying on phenotypic information derived from growth in pure culture and techniques that are time-consuming and difficult to standardize, particularly when compared to the ease of modern high-throughput genome sequencing. Here, drawing on the genus *Acinetobacter* as a test case, we examine whether bacterial taxonomy could abandon phenotypic approaches and DNA-DNA hybridization and, instead, rely exclusively on analyses of genome sequence data.

**Results:**

In pursuit of this goal, we generated a set of thirteen new draft genome sequences, representing ten species, combined them with other publically available genome sequences and analyzed these 38 strains belonging to the genus. We found that analyses based on 16S rRNA gene sequences were not capable of delineating accepted species. However, a core genome phylogenetic tree proved consistent with the currently accepted taxonomy of the genus, while also identifying three misclassifications of strains in collections or databases. Among rapid distance-based methods, we found average-nucleotide identity (ANI) analyses delivered results consistent with traditional and phylogenetic classifications, whereas gene content based approaches appear to be too strongly influenced by the effects of horizontal gene transfer to agree with previously accepted species.

**Conclusion:**

We believe a combination of core genome phylogenetic analysis and ANI provides an appropriate method for bacterial species delineation, whereby bacterial species are defined as monophyletic groups of isolates with genomes that exhibit at least 95% pair-wise ANI. The proposed method is backwards compatible; it provides a scalable and uniform approach that works for both culturable and non-culturable species; is faster and cheaper than traditional taxonomic methods; is easily replicable and transferable among research institutions; and lastly, falls in line with Darwin’s vision of classification becoming, as far as is possible, genealogical.

## Background

In the early eighteenth century, Linnaeus provided the first workable hierarchical classification of species, based on the clustering of organisms according to their phenotypic characteristics [1]. In *The Origin of Species*[2], Darwin added phylogeny to taxonomy, while also emphasizing the arbitrary nature of biological species: “*I look at the term species as one arbitrarily given for the sake of convenience to a set of individuals resembling each other*.” The reality and utility of the species concept continues to inform the theory and practice of biology and a stable species nomenclature underpins the diagnosis and monitoring of pathogenic microorganisms [3-5].

Traditional taxonomic analyses of plants and animals rely on morphological characteristics. However, this approach cannot easily be applied to unicellular microorganisms. In the latter half of the twentieth century, it became clear that bacteria could be grouped into taxonomic clusters based on stable phenotypic characters (e.g. cellular morphology and composition, growth requirements and other metabolic traits) that could be measured reliably in the laboratory. In the 1960s and 1970s, Sneath and Sokal exploited improved technical and statistical methods to develop a numerical taxonomy, which revealed discrete phenotypic clustering within many bacterial genera [6].

Such phenotypic approaches soon faced competition from genotypic approaches, such as DNA base composition (mol% G+C content) [7] and whole-genome DNA-DNA hybridization (DDH); the latter remains the gold standard in bacterial taxonomy [8]. Within this framework, Wayne *et al.*[8] recommended that “a species generally would include strains with approximately 70% or greater DNA-DNA relatedness”. However, few laboratories now perform DNA-DNA hybridization assays as these are onerous and technically demanding when compared to the rapid and easy sequencing of small signature sequences, such as the 16S ribosomal RNA gene. This shift has led to an updated species definition: “a prokaryotic species is considered to be a group of strains that are characterized by a certain degree of phenotypic consistency, showing 70% of DNA–DNA binding and over 97% of 16S ribosomal RNA (rRNA) gene-sequence identity” [9].

Most recently, whole-genome sequencing has delivered new taxonomic metrics—for example, average nucleotide identity (ANI), calculated from pair-wise comparisons of all sequences shared between any two strains. ANI exhibits a strong correlation with DDH values [10], with an ANI value of ≥ 95% corresponding to the traditional 70% DDH threshold [10].

Despite the ready availability of genome sequence data, microbial taxonomy remains a conservative discipline. When defining a bacterial species, most modern microbial taxonomists use a polyphasic approach, whereby a bacterial species represents “a monophyletic and genomically coherent cluster of individual organisms that show a high degree of overall similarity with respect to many independent characteristics, and is diagnosable by a discriminative phenotypic property” [11]. Although the polyphasic approach is pragmatic and widely applicable, it has drawbacks. It relies on phenotypic information, which in turn relies on growth, usually in pure culture, in the laboratory, which may not be achievable for many bacterial species [12]. It also relies on techniques that are time-consuming and difficult to standardize, particularly when compared to the ease of modern genome sequencing [4,13,14].

We, like others, are therefore driven to consider whether, in the genomic era, bacterial taxonomy could, and should, abandon phenotypic approaches and rely exclusively on analyses of genome sequence data [4,10,14-18]. However, such an approach brings fresh conceptual and methodological challenges. Several forces shape the evolution of bacterial genomes: the steady accumulation of point mutations or small insertions/deletions (indels), potentially giving rise to a tree-like phylogeny; the influence of homologous recombination in some lineages, obscuring such diversification; and the key role of gene gain/loss, particularly the pervasive influence of horizontal gene transfer, which, if substantial, could obliterate phylogenetic signals. These forces act with different strength on different parts of the genome and on different bacterial lineages. For example, sequences from a single gene such as the 16S rRNA gene have been shown to fail to capture the true genome-wide divergence between two strains [19-21]. Additionally, it may be expected that the various novel sequence-based metrics would be affected differently by different evolutionary forces. This raises potential problems with the consistency of classification (results may or may not be consistent across the metrics) and backwards compatibility (classification may or may not correspond to already named species within a genus). In this work, we wished to explore these issues on a well-characterized and important bacterial genus, *Acinetobacter*.

The genus *Acinetobacter* was first proposed by Brisou and Prévot in 1954 [22]; however, it was not until Baumann *et al.*[23] published their comprehensive study based on nutritional and biochemical properties that this designation became more widely accepted. In 1974 the genus was listed in *Bergey’s Manual of Systematic Bacteriology* with the description of a single species, *A. calcoaceticus.* To date, there are 27 species described in the genus (http://www.bacterio.cict.fr/a/acinetobacter.html). To fall within genus *Acinetobacter*, isolates must be Gram-negative, strictly aerobic, non-fermenting, non-fastidious, non-motile, catalase-positive, oxidase-negative and have a DNA G+C content of 38-47% [24]. Some isolates within the genus are naturally competent resulting in intra-species recombination [25-27]. Environmental isolates, such as *A. calcoaceticus* PHEA-2 and *Acinetobacter oleivorans* DR1, have attracted interest because they are able to metabolize a diverse range of compounds [28-30]. However, most research on the genus has focused on clinical isolates, particularly from the species *A. baumannii.* This species has shown an astonishing ability to acquire antibiotic resistance genes and some strains are now close to being untreatable [31,32]. Worryingly, the incidence of serious infections caused by other *Acinetobacter* species is also increasing [33]. Genotypic approaches have suggested that *A. baumannii* forms a complex—the *A. baumannii/calcoaceticus* or ACB complex—with three other species *A. calcoaceticus*, *A. nosocomialis* and *A. pittii*. However, it remains very difficult, if not impossible, for a conventional reference laboratory to distinguish these species on phenotypic grounds alone [34]. Techniques such as AFLP and amplified 16S rRNA gene restriction analysis (ARDRA) can be used to identify species within the *Acinetobacter* genus and the ACB complex [35-38]; however, these techniques are too laborious to be carried out in a routine laboratory [24].

Given the general difficulty in defining bacterial species and the ready availability of genome sequence data, we sought to evaluate a range of novel genotypic and genome-based metrics for species delineation. In light of discussed obstacles and the on-going public health concern, we believe that genus *Acinetobacter* provides a timely test case to evaluate the validity and robustness of these sequence-based approaches. In pursuit of this goal, we generated a diverse and informative set of thirteen new draft genome sequences, representing ten species, and we analyzed the whole-genome sequences from a total of 38 strains belonging to the genus.

## Results and discussion

### General genome characteristics

The genomes of thirteen *Acinetobacter* strains, including seven type strains, were sequenced to draft quality using 454 sequencing (Table 1). The *A. bereziniae* strain was found to have the largest genome size within the genus (~ 5 Mb), while the strain with the smallest genome (~2.9 Mb) belonged to the species *A. parvus*, which is known to have a reduced metabolic repertoire compared to other *Acinetobacter* species [39]. These thirteen genomes were considered alongside twenty-five other publicly available genome sequences from the genus *Acinetobacter* (see Additional file 1).

**Table 1:** **Genome sizes, sequencing statistics, G+C content, number of CDSs in the thirteen sequenced *Acinetobacter *isolates**

** Species**	** Strain**	**Genome size (Mb)**	**Peak coverage**	**No. of contigs**	**G+C content (%)**	**No. of predicted good quality CDSs†**	**GenBank accession number**
*A. parvus*	DSM 16617 (T)	2.88	24x	257	41.6	2681	AIEB00000000
*A. radioresistens*	DSM 6976 (T)	3.35	13x	354	41.4	2964	AIDZ00000000
*A. lwoffii*	NCTC 5866 (T)	3.35	14x	260	43.0	3005	AIEL00000000
*A. ursingii*	DSM 16037 (T)	3.57	21x	158	40.0	3252	AIEA00000000
A. pittii*	DSM 21653 (T)	3.75	8x	468	38.8	3252	AIEK00000000
*A. calcoaceticus*	DSM 30006 (T)	3.89	10x	373	38.6	3377	AIEC00000000
*A. baumannii*	W6976	3.91	8x	537	39.0	3252	AIEG00000000
*A. baumannii*	W7282	3.95	14x	140	39.0	3466	AIEH00000000
*A. baumannii*	NCTC 7422	3.99	22x	179	41.3	3626	AIED00000000
A. pittii*	DSM 9306	4.03	11x	339	38.8	3553	AIEF00000000
A. nosocomialis*	NCTC 8102	4.12	10x	283	38.7	3596	AIEJ00000000
A. nosocomialis*	NCTC 10304	4.16	10x	387	39.1	3501	AIEE00000000
*A. bereziniae*	LMG 1003 (T)	4.98	12x	392	38.1	4480	AIEI00000000

* Species names as proposed by Nemec *et al.*[39].

† Definition of good quality CDS is length ≥ 50 codons, of which less than 2% are stop codons.

(T) = Type strain.

#### *A. ursingii* DSM 16037 genome characteristics

The species *A. ursingii* was first described by Nemec *et al.* in 2001 [40]. We have genome sequenced the type strain DSM 16037, which was isolated from a blood culture taken from an inpatient in Prague, Czech Republic in 1993 [40]. In the genome we identified 3252 good-quality CDSs (minimum length 50 codons of which less than 2% are stop codons); 270 of these do not have homologs in any of the other 37 *Acinetobacter* strains in this study. Depth of coverage was generally consistent, apart from two contigs which showed 3.5 times greater-than-average coverage. Scrutiny of the larger of these two contigs (9.4 kb) identified CDSs that are predicted to encode plasmid replication and mobilization proteins. This contig also contains homologs of *sul1* and *uspA* genes, which are often associated with *A. baumannii* resistance islands [41].

#### *A. lwoffii* NCTC 5866 genome characteristics

*A. lwoffii* was first described by Audureau in 1940 under the name *Moraxella lwoffii*[22], but was later moved to genus *Acinetobacter* by Baumann *et al.*[23]. In 1986, Bouvet and Grimont emended the description of the species to designate strain NCTC 5866 the type strain [42]. We identified 3005 good-quality CDSs in the NCTC 5866 genome, of which 229 do not have homologs in any of the *Acinetobacter* genomes examined in this study. Investigation of these CDSs revealed two putative prophages, *ca.* 44.5 and 25.6 kb. Interestingly, many of the CDSs found in these two putative prophages are also present in a recently sequenced environmental *Acinetobacter* strain P8-3-8 (not included in this study) isolated from the intestine of a blue-spotted cornetfish caught in Vietnam [43].

Among the remaining strain-specific CDSs, we identified fourteen that are nearly identical to *tra* genes found in PHH1107, a low GC content plasmid isolated from pig manure [44]. The *tra* homologs are distributed on two contigs, one of which has a GC content (37%) lower than the genome mean (43%).

#### *A. parvus* DSM 16617 genome characteristics

Strain DSM 16617 is the type strain for *A. parvus* isolated from the ear of an outpatient from Pribram, Czech Republic in 1996 [45]. We identified 2681 good-quality CDSs in the DSM 16617 genome, 179 of which do not have homologs in any of the remaining 37 genomes. Analysis with Prophinder [46] identified one 39kb putative prophage containing phage-related genes homologs to putative phage-related genes found in *A. baumannii* and *A. oleivorans* DR1. We identified an 8kb contig with 2.5 times higher than average depth of coverage, which contains homologs to phage related genes.

#### *A. bereziniae* LMG 1003 genome characteristics

Strain LMG 1003 is the type strain for *A. bereziniae*, a recently named species by Nemec *et al.*, which has been isolated from various human, animal and environmental sources [47]. We identified 4480 good-quality CDSs in the genome, with 1061 strain-specific CDSs (no homologs in the rest of the 37 genomes). This is a considerably higher percentage, 24%, than in other *Acinetobacter* strains (see Additional file 1). Many of the strain-specific CDSs form clusters of four or more CDSs, with the largest cluster containing 49 consecutive CDSs, of which 45 are strain-specific. Twenty-one CDSs in this cluster have no significant similarity to proteins in the non-redundant protein database.

Depth of coverage analysis revealed several contigs with higher than average value. One such contig has 5 times greater coverage compared to the rest of the genome, which suggests it is a mobile element. It contains a CDS homologous to the *sul1* gene often found in *A. baumannii* resistance islands [41].

#### *A. radioresistens* DSM 6976 genome characteristics

*A. radioresistens* strain DSM 6976 was isolated in 1979 from cotton sterilized by γ-radiation and is the type strain for the species [48]. We identified 2964 good-quality CDSs in the genome, of which 188 do not have homologs in any of the remaining 37 genomes.

A comparison with two previously sequenced *A. radioresistens*, SK82 and SH164, reveals that the three strains share 2458 CDSs (about 83% of the average number of CDSs in these three strains), 43 of which were not found in the remaining 35 *Acinetobacter* genomes. Among these there is a homolog of the *metE* gene, and two genes involved in the degradation of benzoate, an aromatic compound which is known to support the growth of a number of *A. radioresistens*[49]. Though the three strains are quite similar, we identified 143 CDSs in DSM 6976 which are absent in SK82 and SH164, but do have homologs in other *Acinetobacter* genomes. Within this group there is a genomic island containing nine genes related to fructose metabolism and a cluster of four CDSs predicted to encode for type IV pilin proteins.

### Phylogenetic relationships within genus *Acinetobacter*

**Figure 1:**
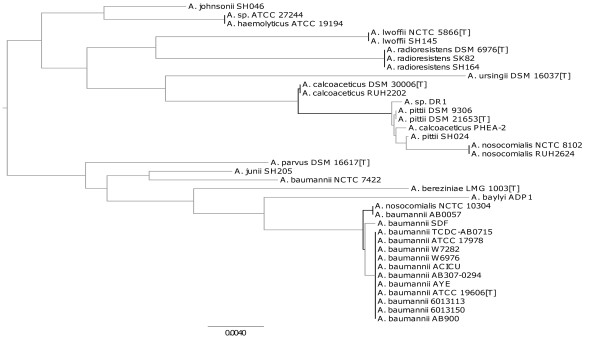
**Phylogenetic tree based on the 16S rRNA gene sequences. **The tree was built for 37 *Acinetobacter* isolates (*A. baumannii* 6014059 was excluded as only partial 16S sequence was identified) and rooted at midpoint. Outgoing branches of a node are depicted in black if bootstrap support (100 replicates) at the node is ≥ 70%; in grey otherwise. The tree is significantly divergent from previous published results, e.g. the monophyly of the ACB complex is not preserved.

Stackebrandt and Goebel suggested that bacterial species can be delineated using 16S rRNA gene sequences: according to their criteria, when two aligned sequences exhibit ≥ 97% identity, the isolates from which they originate are deemed to belong to the same species [50]. However, when we extracted 16S rRNA gene sequences from the *Acinetobacter* genomes in this study, we found that these criteria gave inconsistent results. For example, the 16S rRNA genes from the type strains of *A. baumannii* and *A. radioresistens* exhibit 97% sequence identity, suggesting they should be in the same species. Similarly, sequences from the type strains of *A. calcoaceticus* and *A. lwoffii* show 97.6% identity, again suggesting they should be classified in the same species. Recent studies by Keswani and Whitman [51] and Stackebrandt and Ebers [52] have suggested a revised cut-off value of ≈ 99% 16S rRNA identity for species delineation. We found that even using this stricter cut-off, we were not able to find evidence for delineating the type strains of *A. calcoaceticus* and *A. pittii* (99.3%), and the type strain of *A. pittii* from *A. nosocomialis* strains NCTC 8102 and RUH2624 (99.5%). Furthermore, when a phylogenetic tree is constructed from 16S rRNA sequence data, the monophyly of the ACB complex was not preserved and the confidence values for most branches fall below 70% (Figure 1). Similar problems with using 16S rRNA gene sequences to resolve species have been reported in other genera [11,21].

**Figure 2:**
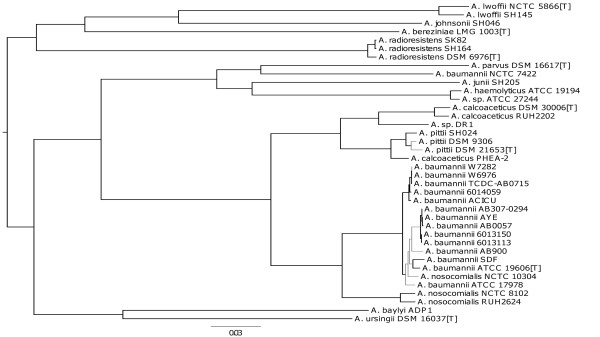
**Phylogenetic tree based on 127 CDSs present in all 38 strains. **The 127 CDSs used for this tree are present in all strains, have no paralogs and show no signs of recombination. The tree is rooted at midpoint. Outgoing branches of a node are depicted in black if bootstrap support (100 replicates) at the node is ≥ 70%; in grey otherwise.

Given the highly conserved nature of the 16S rRNA gene sequences, we attempted to reconstruct a phylogeny based on more comprehensive gene set -- the core genome of the genus. We found 911 orthologous coding sequences (CDSs) present in all thirty-eight strains, representing around a quarter of the average number of CDSs per strain. However, concerned that naïve use of this dataset might lead to problems due to homologous recombination, we selected a subset of 127 single-copy CDSs that showed with no signs of recombination according to three different measures (see Methods). These were concatenated, aligned and used to derive a phylogenomic tree (Figure 2). Interestingly, a tree constructed with no recombination filtering was nearly identical to the tree based on recombination-free CDSs (see Additional file 2).

This core genome tree generally supports the monophyletic status of the named species within the genus, with three exceptions: *A. baumannii* NCTC 7422 belongs in a deep-branching lineage with the *A. parvus* type strain DSM 16617, *A. nosocomialis* NCTC 10304 clusters within *A. baumannii* and *A. calcoaceticus* PHEA-2 is closer to the three *A. pittii* strains than to the other two *A. calcoaceticus* strains. The first two strains have been genome-sequenced as part of this study and our results suggest they have been misclassified in the culture collection. PHEA-2 is an isolate from industrial wastewater that was genome-sequenced by Xu *et al.*[53]. Our core genome tree and comparisons of 16S rRNA gene sequences show PHEA-2 to be closer to the three *A. pittii* strains than to the other two *A. calcoaceticus* strains, suggesting it too has been misclassified. Interestingly, the previously unclassified strain DR1 sits closest to the two *A. calcoaceticus* strains, while ATCC 27244 is closest to the species *A. haemolyticus*.

Once such reclassifications are taken into account, our core genome phylogenetic tree is consistent with the currently accepted genus taxonomy and also supports the monophyly of the ACB complex and of each of its four constituent species. Within *A. baumannii*, two lineages, international clones I and II, previously identified by comparative cell envelope protein profiling, ribotyping and AFLP genomic fingerprinting [53] are present as monophyletic groups in our tree. The tree obtained from the core genome is similar to a tree obtained from a recently described approach based on 42 ribosomal genes [15] (see Additional file 3).

### Rapid genomic approaches to species delineation

Phylogenetic approaches are processor-intensive. We therefore evaluated genetic relatedness among the 38 strains using three rapid distance-based oligonucleotide and gene content approaches that avoid time-consuming calculations: the previously mentioned ANI, as well as K-string [54] and genome fluidity [55] approaches.

**Figure 3:**
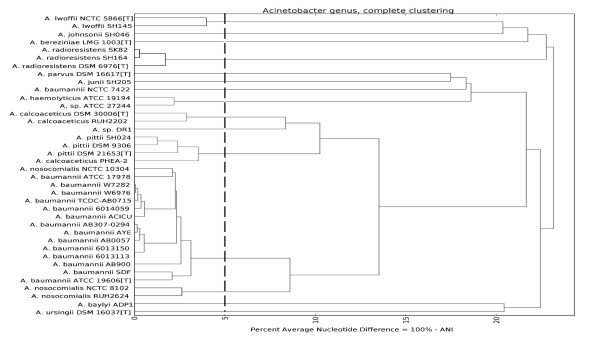
**The Average Nucleotide Identity (ANI) dendogram for the 38 strains. **The vertical dashed line represents the 95% species cutoff value proposed by Goris *et al.* (10).

ANI relies on the identification of alignable stretches of nucleotide sequence in genome pairs, followed by a scoring and averaging of sequence identity, ignoring any divergent regions. The topology of the dendogram based on ANI analysis (Figure 3) is congruent with our core genome phylogenetic tree, confirming the misclassifications and new relationships already identified, while also showing the two international clones as separate lineages within *A. baumannii*.

The K-string composition approach [54] is based on oligopeptide content analysis of predicted proteomes. The divergence dendogram for K=5 (see Additional file 4) generally agrees with the results from the phylogenetic tree and ANI dendogram at species level. However, the major problem is that the K-string approach places *A. baumannii* SDF outside the ACB complex, probably reflecting the considerable difference in gene repertoires between this drug-sensitive strain and all other genome-sequenced *A. baumannii* strains.

Genome fluidity provides a measure of the dissimilarity of genomes evaluated at the gene level [55]. A dendogram based on genomic fluidity (see Additional file 5) significantly differs from the results obtained with other techniques: *A. baumannii* SDF again sits outside the ACB complex, *A. nosocomialis* strains NCTC 8102 and RUH2624 now sit within the *A. baumannii* clade and PHEA-2 sits not with the *A. pittii* strains but with DR1 and the other *A. calcoaceticus* strains. We also performed pair-wise comparison of the gene content of the 38 strains, calculating the amount of the CDSs shared by each pair of strains (see Additional file 6). While strains from the same species generally share at least 80% of their CDSs, we found strains from different species exhibiting similar ratios. For example, *A. calcoaceticus* RUH2202 shares more than 80% of its CDS repertoire with DR1 and various *A. nosocomialis*, *A. baumannii*, *A. pittii* strains; PHEA-2 and DR1 share 88.1% of their CDSs. Based on gene content only, *A. baumannii* SDF is distinct from all other *A. baumannii* strains in our study (sharing at most 71.6% of its CDSs), which explains its placement in the K-string and genomic fluidity dendograms (see Additional files 4 and 5, respectively). These results indicate a potentially significant level of horizontal gene transfer among *Acinetobacter* species and illustrate an inability to delineate species based on gene content comparison only.

These findings suggest that ANI analyses provide results that are compatible with traditional and phylogenetic classifications, whereas K-string and genome fluidity approaches appear to be too strongly influenced by the effects of horizontal gene transfer to be consistent with previously accepted approaches.

### Defining species in *Acinetobacter* on the basis of whole-genome analyses

The congruence of the phylogenetic tree and ANI dendogram with each other and with existing species definitions provides confidence that these techniques are fit for purpose in delineating species in the absence of phenotypic data. Furthermore, as Goris *et al.* suggest, the ANI approach provides a handy numerical cut-off at 95% identity to demarcate species boundaries, which corresponds to the 70% DDH value [10]. When we applied this cut-off to our dataset, we were able to classify 37 of the strains into thirteen previously named species.

In line with the likely misclassification of strains, we observed that *A. nosocomialis* NCTC 10304 shares phylogenetic history and exhibits pair-wise ANI values greater than 95% with all 14 sequenced *A. baumannii* strains, thus confirming it should be designated *A. baumannii* NCTC 10304. Similar arguments apply for *A. calcoaceticus* PHEA-2 (new designation *A. pittii* PHEA-2) and *A. sp.* ATCC 27244 (*A. haemolyticus* ATCC 27244). However, the strain NCTC 7422 appears to be distinctive enough to represent new species. While the traditional polyphasic approach to taxonomy demands additional phenotypic characterization before these species can be named, on the basis of the analyses presented here, we propose the species name ***Acinetobacter bruijnii*** sp. nov. (N. L. gen. masc. n. bruijnii, of Bruijnius, named after Nicolaas Govert de Bruijn, Dutch mathematician) for strain NCTC 7422 and all future strains that are monophyletic and show ≥ 95% ANI to this strain.

It is interesting to note that our results based on core genome and ANI analyses differ from those based on AFLP patterns [56]; notably in the latter *A. haemolyticus* and *A. junii* do not cluster together nor does the cluster form a sister branch to the ACB complex; also *A. johnsonii* does not appear on the same deep-branch as *A. lwoffii*. This observation suggests that although AFLP is adept at species resolution, it appears to be unsuitable for phylogenetic analysis.

Several recent studies report alternative genomic approaches to bacterial taxonomy and species identification. These include *in silico* multilocus sequence analysis (MLSA), average amino acid identity (AAI) and ribosomal multilocus sequence typing (rMLST), which have been used to delineate species in the genera *Neisseria*, *Vibrio* and *Mycoplasma*[17,18,57]. Although MLSA can be used to infer phylogeny, this approach suffers from arbitrariness in choice of in genes which varies from one taxon to the next. Our proposed approach, core-genome phylogeny, can be considered an extension of MLSA and rMLST. However, as it is based on all shared CDSs in a given genus, it makes use of all potentially informative sequence sites. ANI, like AAI, measures pair-wise similarities between genome sequences but provides better resolution of species and sub-species [58,59].

## Conclusions

The aim of this study has been to determine, using the genus *Acinetobacter* as a test case, whether genome sequence data alone are sufficient for the delineation and even definition of bacterial species. To this end, we explored the applicability of two broad approaches: sequence-based phylogenies for single and multiple gene and distance-based methods that include gene content comparisons (K-string and genomic fluidity) and whole-genome sequence similarities (ANI). We have found that a phylogenetic analysis of the genus *Acinetobacter* based on 16S rRNA gene sequences provides unreliable and uninformative results. By contrast, a core genome phylogenetic tree provides robust, informative results that are backwards compatible with the existing taxonomy.

Among the distance metrics, we found that approaches using gene content (K-string and genomic fluidity) led to anomalous conclusions, e.g., placing the SDF strain outside of the *A. baumannii* cluster, presumably because they are affected by horizontal gene transfer. In contrast, the easy-to-compute ANI results are congruent with the core genome phylogeny and traditional approaches. Using the core genome phylogeny and ANI approach, we found three misclassifications, one of which represents new species. These findings illustrate the need to genome-sequence all strains archived in culture collections, which is likely to become technically and economically feasible in the near future.

We believe a combination of core genome phylogenetic analysis and ANI provides a feasible method for bacterial species delineation, in which species are defined as monophyletic groups of isolates that exhibit at least 95% pair-wise ANI to each other. This approach combines a theoretically rigorous approach (sequence phylogeny) with a pragmatic metric (ANI) that provides a numerical cut-off that is backwards compatible and has been shown to be applicable to a diverse group of bacteria [10,60].

Our sequence-based approach has several desirable characteristics. Firstly, it is capable of resolving the inconsistency in classification of genomospecies. For example, our results confirm the recent assignment of genomospecies 3 and 13TU to Latin binomials *A. pittii* and *A. nosocomialis*, respectively. Secondly, it provides a scalable and uniform approach that works for both culturable and non-culturable species, solving the problem in classifying non-culturable organisms, in an era when whole-genome sequences of such organisms can be recovered relatively easily via metagenomics or single-cell genomics. Thirdly, our approach is faster and cheaper than traditional taxonomic methods, as well as being easily replicable and transferable among research institutions. Finally a method that combines phylogeny and pragmatism falls in line with Darwin’s vision of classification, as stated in the conclusion of Origin of Species: “*Our classification will come to be, as far as they can be so made, genealogies…*” [2].

## Methods

### Strain selection and growth conditions

Details of *Acinetobacter* strains used in this study are listed in Additional file 1. *Acinetobacter baumannii* W6976 and W7282 were provided by Drs. Mike Hornsey and David Wareham at Barts and The London NHS Trust, whilst the remaining strains were obtained from the UK, German and Belgium culture collections. Sequenced isolates were cultured in Nutrient broth or Tryptic soy medium at 25°C or 30°C. DNA was extracted from single colony cultures using Qiagen 100/G Genomic-tips and quantified using Quant-iT PicoGreen dsDNA kits (Invitrogen). DNA was stored at 4°C.

### Genomic sequencing and annotation

DNA from thirteen isolates was sequenced by 454 GS FLX pyrosequencing (Roche, Branford, CT, USA) according to the standard protocol for whole-genome shotgun sequencing, producing an average of 450bp fragment reads. Draft genomes were assembled from flowgram data using Newbler 2.5 (Roche). The resulting contigs were annotated using the automated annotation pipeline on the xBASE server [61]. The genome sequences of the thirteen newly sequenced strains have been deposited in GenBank as whole genome shotgun projects (Table 1).

### Ortholog computation

We computed the set of all orthologs within the 38 strains in our study with OrthoMCL [62] which performs a bidirectional best hit search in the amino-acid space, followed by a subsequent clustering step (percentMatchCutoff = 70, evalueCutoff = 1e-05, I = 1.5). Predicted are 7,334 clusters of orthologous groups (COGs) containing 124,870 coding sequences (CDSs), which represents 95.7% of all good-quality CDSs (length at least 50 codons of which less than 2% are stop codons).

### Core genome phylogenetic tree construction

Using the orthologs data, we extracted the genus core genome, i.e. the set of COGs which are present in each of the 38 strains (911 COGs). We filtered this set to exclude COGs containing paralogs and obtained a set of 827 single-copy COGs. The nucleotide gene sequences of each single-copy COG were aligned using MUSCLE 3.8.31 [63] with default parameters and the alignments were trimmed for quality, leading and trailing blocks using GBlocks 0.91b [64] with default parameters. After excluding 8 COGs with trimmed length < 50 bp, we screened the remaining 819 COGs for possible evidence of recombination using the PHI [65], MaxChi [66] and Neighbour similarity score [67] tests implemented in PhiPack (http://www.maths.otago.ac.nz/~dbryant/software/PhiPack.tar) using 1000 permutations, window size = 50 bp and p-value < 0.05. To facilitate a more robust phylogeny construction, we selected only the 127 recombination-free COGs for which none of the three tests found evidence of recombination. The trimmed alignments of the 127 COGs were concatenated and used to build the tree by the approximately maximum-likelihood FastTree 2 [68] with 100 bootstrap replicates (created using SEQBOOT program from the PHYLIP package [69]. The resulting tree was visualized using FigTree (http://tree.bio.ed.ac.uk/software/figtree) and rooted at the mid-point.

The trees based on the 16S, the 819 single-copy COGs (no recombination filtering) and the 42 ribosomal genes were built in the same manner – multiple alignment of the nucleotide sequences with MUSCLE, trimming with GBlocks, and constructing bootstrapped trees (100 replicates) with FastTree 2, rooting them at mid-point.

### Average nucleotide identity (ANI)

The ANI analysis was based on whole-genome data using the method proposed by Goris *et al.*[10]. Briefly, for each genome pair, one of the genomes was chosen as a query and split into consecutive 500 bp fragments. These were then used to interrogate the second genome, designated the reference, using BLASTn [70] (X = 150, q = -1 F= F). For each query, the hit with the highest bit-score was selected and if the alignment exhibited at least 70% identity and over 70% of the query fragment length, the hit was retained for further evaluation. The ANI score was computed as the mean identity of the retained hits. Based on the pair-wise ANI values, we compiled a distance matrix to represent the ANI divergence (which is defined as 100% - ANI) between the strains and used it to compute the ANI divergence dendogram with the hierarchical clustering package hcluster 0.2.0 adopting the complete linkage algorithm (http://pypi.python.org/pypi/hcluster).

### Gene repertoire comparison (K-string and genomic fluidity)

K-string analysis was based on the method proposed by Qi *et al.*[54]; for each proteome, its composition vector was computed by extracting the frequency of overlapping amino acid strings of length K and filtering out the random mutation background using a Markov model. The divergence between two genomes was computed by calculating the cosine function of the angle between the pair’s composition vectors. The dendogram based on the pair-wise K-string distances was built as for ANI. The pair-wise genomic fluidity for each pair of genomes was computed using the ortholog data as suggested by Kislyuk *et al.*[55]. The dendogram was built as for ANI and K-string.

## Authors’ contributions

JC and MH designed and performed the study, analyzed data, drafted and revised the manuscript. NL analyzed data and revised the manuscript. CC performed the whole-genome sequencing and revised the manuscript. MP conceived and designed the study and revised the manuscript. All authors read and approved the final manuscript.

## Supplementary Material

Additional file 1 **The 38 sequenced *Acinetobacter *strains used in this study.** Click here for file

Additional file 2 Phylogenetic tree based on 819 core CDSs (without recombination filtering). Click here for file

Additional file 3 **Phylogenetic tree based on 42 ribosomal genes (Jolley ***et al. *) [15]. Click here for file

Additional file 4 **K-string analysis of the 38 *Acinetobacter *strains used in this study.** Click here for file

Additional file 5 **Genomic fluidity analysis of the 38 *Acinetobacter *strains used in this study.** Click here for file

Additional file 6 **Pair-wise gene content comparison of the 38 *Acinetobacter *strains used in this study.** Click here for file
