# Supplementary material for: Defining bacterial species in the genomic era: insights from the genus Acinetobacter
Source: BMC Microbiol. 2012 Dec 23;12:302. doi: 10.1186/1471-2180-12-302 (PMC3556118; doi:10.1186/1471-2180-12-302)
Supplement: Additional file 1 — The 38 sequenced Acinetobacter strains used in this study. [file 1471-2180-12-302-S1.pdf]

Supplementary Table 1. The 38 sequenced *Acinetobacter* strains used in this study.

Species	Strain	Accession no. [1]	Isolation source	Geographical location	Isolation date	Status	Percentage strain-specific CDSs (within genus)	Reference
A. baumannii	6013113	ACYR00000000*	Skin	Selly Oak Hospital, UK	2007	WGS	0.2%	The Genome Institute at Washington University[2]
A. baumannii	6013150	ACYQ00000000*	Skin	Selly Oak Hospital, UK	2007	WGS	0.2%	The Genome Institute at Washington University[2]
A. baumannii	6014059	ACYS00000000*	Skin	Selly Oak Hospital, UK	2007	WGS	1.0%	The Genome Institute at Washington University[2]
A. baumannii	AB0057	NC_011586 (NC_011585)	Blood	WRAMC, USA	2004	Complete	1.4%	(4)
A. baumannii	AB307-0294	NC_011595	Blood	Buffalo, NY, USA	1994	Complete	0.7%	(4)
A. baumannii	AB900	ABXK00000000*	Perineum	WRAMC, USA	2003	Draft	3.7%	(4)
A. baumannii	ACICU	NC_010611 (NC_010605, NC_010606)	CF fluid	Rome, Italy	2005	Complete	1.9%	(5)
A. baumannii	ATCC 17978	NC_009085 (NC_009083, NC_009084)	Meningitis	Unknown	1951	Complete	1.1%	(6)
A. baumannii	ATCC 19606 (T)	ACQB00000000*	Urinary tract infection	USA	1948	WGS	3.1%	Broad Institute[3]
A. baumannii	AYE	CU459141 (CU459137, CU459138, CU459140, CU459139)	Pneumonia & urine infection	France	2001	Complete	1.3%	(7)
A. baumannii	NCTC 7422	AIED00000000*	Leg burn	Unknown	1968	WGS	8.5%	This study
A. baumannii	SDF	CU468230 (CU468231, CU468232, CU468233)	Human Louse	France	2000	Complete	4.8%	(7)
A. baumannii	TCDC-AB0715	CP002522 (CP002523, CP002524)	Clinical isolate	Taiwan	2007-2009	Complete	0.7%	(8)
A. baumannii	W6976	AIEG00000000*	Clinical isolate from line tip	London, UK	2009	WGS	0.1%	This study
A. baumannii	W7282	AIEH00000000*	Clinical isolate from intra-abdominal drain fluid post-tigecycline treatment	London, UK	2009	WGS	0.1%	This study
A. baylyi	ADP1	NC_005966	Mutant of a soil isolate	Unknown	1969	Complete	11.0%	(9)
A. bereziniae	LMG 1003 (T)	AIEI00000000*	Wound	Unknown	1966	WGS	23.7%	This study
A. calcoaceticus	DSM 30006 (T)	AIEC00000000*	Quinate enrichment from soil	Unknown	1911	WGS	3.1%	This study
A. calcoaceticus	PHEA-2	CP002177	Industrial wastewater	China	2000	Complete	1.2%	(10, 11)
A. calcoaceticus	RUH2202	ACPK00000000*	Human skin wound	Malmo, Sweden	Unknown	WGS	2.5%	Broad Institute[3]
A. haemolyticus	ATCC 19194	ADMT00000000*	Nose	Unknown	Unknown	Draft	6.8%	HMP
A. johnsonii	SH046	ACPL00000000*	Perineum	Unknown	1993	WGS	11.1%	Broad Institute[3]
A. junii	SH205	ACPM00000000*	Perineum	Unknown	1994	WGS	6.6%	Broad Institute[3]

A. lwoffii	NCTC 5866 (T)	AIEL00000000*	Unknown	Paris	1939	WGS	7.6%	This study
A. lwoffii	SH145	CM001194	Hand	Unknown	1994	WGS	7.3%	Broad Institute[3]
A. nosocomialis	NCTC 8102	AIEJ00000000*	Fecal specimens	Rhode Island, USA	1944	WGS	3.2%	This study
A. nosocomialis	NCTC 10304	AIEE00000000*	Conjunctiva	Unknown	1962	WGS	4.1%	This study
A. nosocomialis	RUH2624	ACQF00000000*	Human skin swab	Unknown	Unknown	Draft	2.4%	HMP
A. oleivorans	DR1	CP002080	Rice paddy soil	Deok-So, South Korea	2010	Complete	6.7%	(12)
A. parvus	DSM 16617 (T)	AIEB00000000*	Human ear	Pribram, Czech Republic	1996	WGS	1.9%	This study
A. pittii	DSM 9306	AIEF00000000*	Urine	Unknown	1990	WGS	1.1%	This study
A. pittii	DSM 21653 (T)	AIEK00000000*	Cerebrospinal fluid	Unknown	Unknown	WGS	1.6%	This study
A. pittii	SH024	ADCH00000000*	Skin	Unknown	Unknown	Draft	6.3%	Broad Institute[3]
A. radioresistens	DSM 6976 (T)	AIDZ00000000*	Cotton tampon sterilized by gamma radiation	Unknown	1979	WGS	1.7%	This study
A. radioresistens	SH164	ACPO00000000*	Human forehead	Unknown	1994	Draft	3.8%	Broad Institute[3]
A. radioresistens	SK82	ACVR00000000*	Human skin	Unknown	Unknown	Draft	5.6%	HMP
Unnamed	ATCC 27244	ABYN01000000	Cervix	Unknown	Unknown	WGS	4.6%	HMP
A. ursingii	DSM 16037 (T)	AIEA00000000*	Blood	Unknown	1993	WGS	8.3%	This study

* Genome project accession number.
[1] The accession numbers of the plasmids belonging to their associated strains are shown in parentheses
[2] Further information can be found at http://genome.wustl.edu/genomes/category/microorganisms/
[3] Further information can be found at www.broadinstitute.org/annotation/genome/Acinetobacter_group/GenomeDescriptions.html
HMP = Human Microbiome Project
