# Supplementary material for: Defining bacterial species in the genomic era: insights from the genus Acinetobacter
Source: BMC Microbiol. 2012 Dec 23;12:302. doi: 10.1186/1471-2180-12-302 (PMC3556118; doi:10.1186/1471-2180-12-302)
Supplement: Additional file 2 — Phylogenetic tree based on 819 core CDSs (without recombination filtering). [file 1471-2180-12-302-S2.pdf]

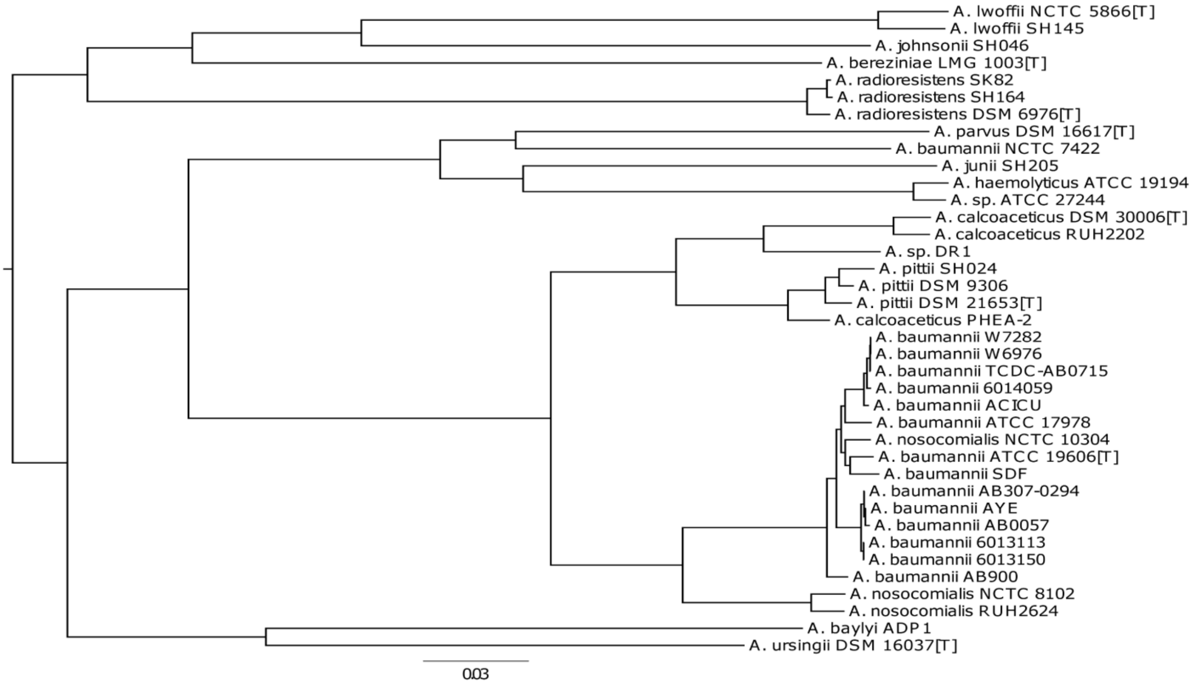

Additional File 2. Phylogenenetic tree based on 819 CDSs present in all 38 strains. The tree is rooted at midpoint. All branch nodes have ≥ 70% bootstrap support (100 replicates). These 819 CDSs are present in all strains, do not contain paralogs, but no recombination filtering step was applied in contrast to Figure 2 in the main manuscript. The main difference between the result here and that presented in Figure 2 is the position of the SDF, ATCC 19606, NCTC 10304 and ATCC 17978 strains – here they sit closer to International Clone II, whilst in Figure 2 they are closer to International Clone I. However, at species level the two approaches (with/without recombination filtering) provide similar results, suggesting that gene recombination in genus *Acinetobacter* is mainly confined within species.
