# Supplementary material for: Defining bacterial species in the genomic era: insights from the genus Acinetobacter
Source: BMC Microbiol. 2012 Dec 23;12:302. doi: 10.1186/1471-2180-12-302 (PMC3556118; doi:10.1186/1471-2180-12-302)
Supplement: Additional file 3 — Phylogenetic tree based on 42 ribosomal genes (Jolley et al. ) [15]. [file 1471-2180-12-302-S3.pdf]

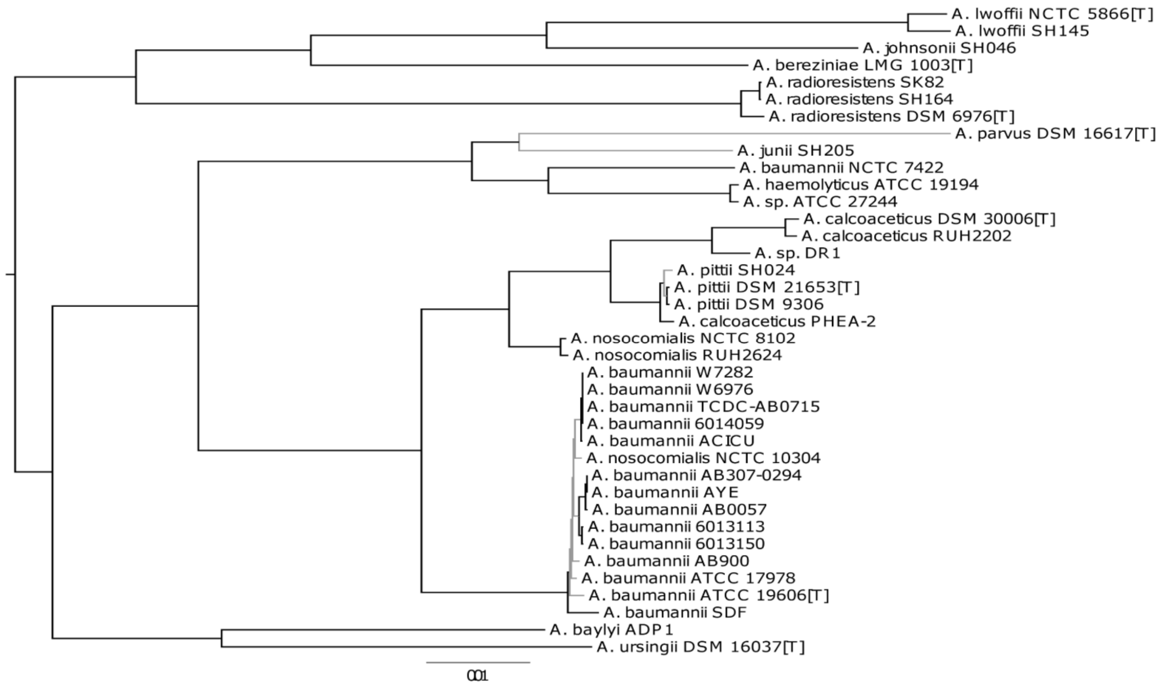

Additional File 3. Phylogenetic tree based on 31 of 42 ribosomal genes suggested by Jolley *et al.* (15) to be used for phylogenomic reconstruction. The tree is rooted at midpoint. Outgoing branches of a node are depicted in black if bootstrap support (100 replicates) at the node is ≥ 70%; in grey otherwise. Out of the 11 ribosomal genes for which a full match was not found in all 38 strains, 9 are missing in up to three strains (S2, S7, S17, S18, S19, S21, L28, L31, L35) possibly due to the WGS quality of some of the strains; L34 was found in only 10 strains and L36 was not found in any strain. The main difference with previous results is the placement of *A. nosocomialis* strains closer to *A. pittii* and *A. calcoaceticus*, rather than with *A. baumannii* as seen before.
