# Supplementary material for: Defining bacterial species in the genomic era: insights from the genus Acinetobacter
Source: BMC Microbiol. 2012 Dec 23;12:302. doi: 10.1186/1471-2180-12-302 (PMC3556118; doi:10.1186/1471-2180-12-302)
Supplement: Additional file 4 — K-string analysis of the 38 Acinetobacter strains used in this study. [file 1471-2180-12-302-S4.pdf]

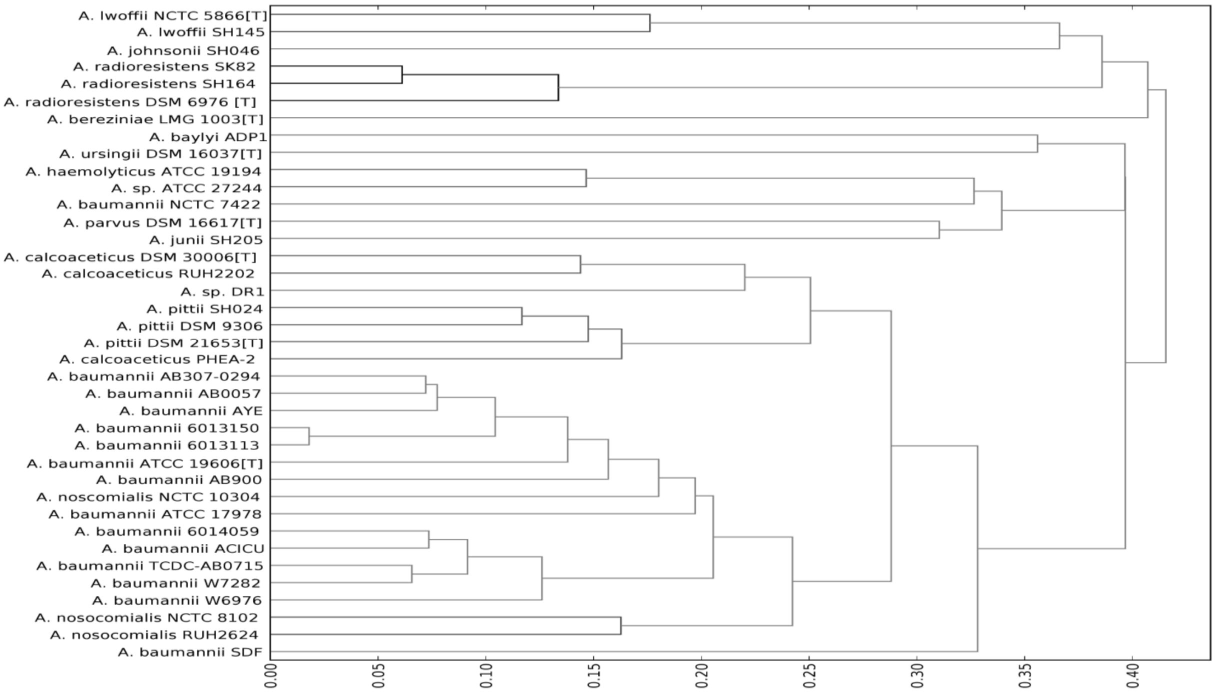

Additional File 4. Relationship dendogram of the 38 strains based on the alignment-free K-string analysis proposed by Qi *et al*. [54] with k = 5. *A. baumannii* SDF is placed outside the ACB complex. This is probably due to the difference in CDS repertoires between this drug-sensitive strain and all other genome-sequenced *A. baumannii*.
