# Supplementary material for: Defining bacterial species in the genomic era: insights from the genus Acinetobacter
Source: BMC Microbiol. 2012 Dec 23;12:302. doi: 10.1186/1471-2180-12-302 (PMC3556118; doi:10.1186/1471-2180-12-302)
Supplement: Additional file 5 — Genomic fluidity analysis of the 38 Acinetobacter strains used in this study. [file 1471-2180-12-302-S5.pdf]

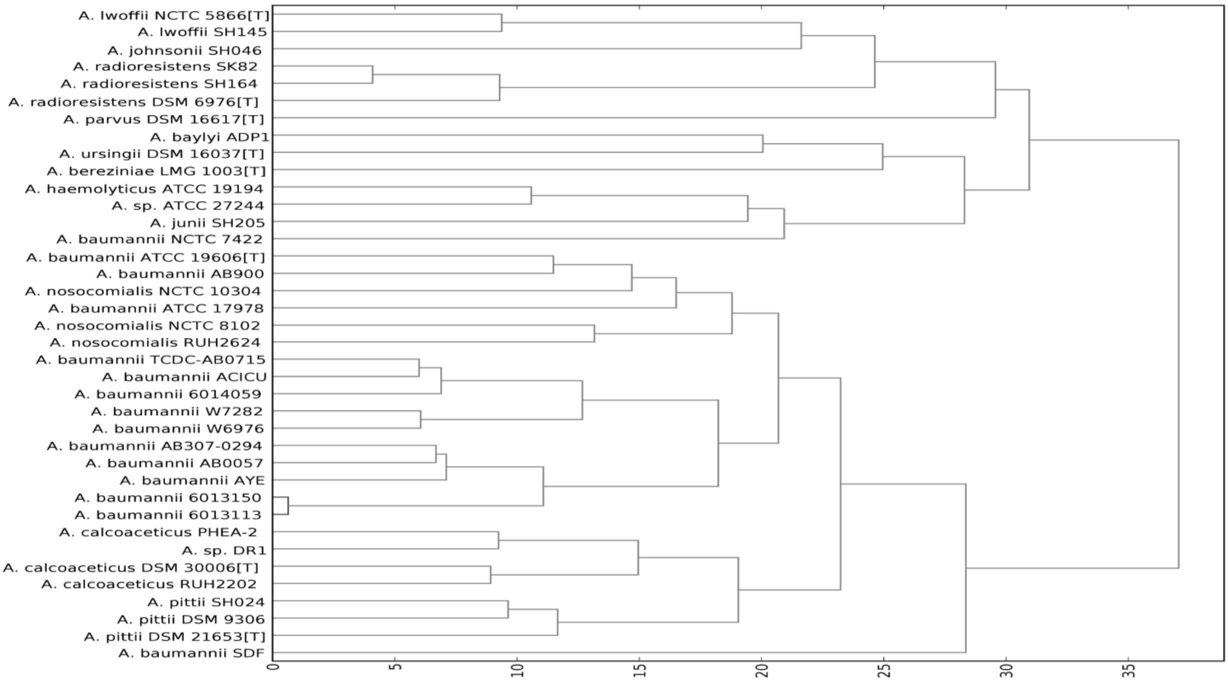

Additional File 5. Relationship dendogram of the 38 strains based on the genomic fluidity analysis proposed by Kislyuk *et al.* [55]. *A. baumannii* SDF is again outside the ACB complex, the two *A. nosocomialis* strains fall within the *A. baumannii* cluster and PHEA-2 is closest to DR1 and the two *A. calcoaceticus* strains. These differences may be explained by the fact that genomic fluidity is strongly affected by putative horizontal gene transfer events.
