# Supplementary material for: Defining bacterial species in the genomic era: insights from the genus Acinetobacter
Source: BMC Microbiol. 2012 Dec 23;12:302. doi: 10.1186/1471-2180-12-302 (PMC3556118; doi:10.1186/1471-2180-12-302)
Supplement: Additional file 6 — Pair-wise gene content comparison of the 38 Acinetobacter strains used in this study. [file 1471-2180-12-302-S6.pdf]

	A. ursingii DSM 16037 (T)	*A. baylyi* ADP1	*A. nosocomialis* RUH2624	*A. nosocomialis* NCTC 8102	*A. baumannii* ATCC 17978	*A. nosocomialis* NCTC 10304	*A. baumannii* ATCC 19606 (T)	*A. baumannii* SDF	*A. baumannii* AB900	*A. baumannii* 6013113	*A. baumannii* 6013150	*A. baumannii* AB0057	*A. baumannii* AYE	*A. baumannii* AB307-0294	*A. baumannii* ACICU	*A. baumannii* 6014059	*A. baumannii* TCDC-AB0715	*A. baumannii* W6976	*A. baumannii* W7282	*A. calcoaceticus* PHEA-2	*A. pittii* DSM 21653 (T)	*A. pittii* DSM 9306	*A. pittii* SH024	*A. sp.* DR1	*A. calcoaceticus* RUH2202	*A. calcoaceticus* DSM 30006 (T)	*A. sp.* ATCC 27244	*A. haemolyticus* ATCC 19194	*A. junii* SH205	*A. baumannii* NCTC 7422	*A. parvus* DSM 16617 (T)	*A. radioresistens* DSM 6976 (T)	*A. radioresistens* SH164	*A. radioresistens* SK82	*A. bereziniae* LMG1003 (T)	*A. johnsonii* SH046	*A. lwoffii* SH145
A. lwoffii NCTC 5866 (T)	68	65	65	63	65	60	63	63	63	63	63	62	64	65	64	64	62	61	63	64	63	64	64	62	64	62	66	66	68	63	66	71	74	72	60	72	84
A. lwoffii SH145	69	65	66	63	64	60	64	65	63	63	63	63	65	65	63	64	62	61	63	64	63	64	65	62	64	61	68	67	70	64	68	73	75	73	61	75	
A. johnsonii SH046	72	67	68	66	68	63	66	67	66	66	66	65	67	67	67	66	65	64	66	68	66	66	68	65	68	64	70	69	72	67	68	71	72	71	64		
A. bereziniae LMG1003 (T)	64	64	64	63	64	60	64	60	63	62	62	62	64	64	63	63	62	60	63	65	61	63	64	63	64	61	62	61	63	65	57	61	63	62			
A. radioresistens SK82	70	69	68	68	69	65	68	65	68	68	68	68	69	70	68	69	67	66	68	69	68	68	69	66	69	66	67	67	68	67	66	86	94				
A. radioresistens SH164	72	70	70	68	71	66	70	67	69	69	69	69	70	72	70	70	68	67	70	71	69	69	70	68	70	67	68	68	70	68	68	87					
A. radioresistens DSM 6976 (T)	70	69	67	66	69	65	68	66	67	67	67	67	68	69	68	68	66	65	67	69	67	66	68	65	68	65	67	66	68	66	66						
A. parvus DSM 16617 (T)	68	64	63	61	63	59	62	65	62	61	61	61	62	63	61	62	60	60	61	62	61	61	62	60	62	59	71	69	73	65							
A. baumannii NCTC 7422	69	70	72	69	71	67	70	64	70	70	70	70	70	71	70	70	69	67	70	72	70	70	72	71	72	67	74	73	75								
A. junii SH205	71	68	71	69	69	66	68	66	69	68	68	67	69	69	67	68	66	66	68	69	68	69	70	67	70	66	76	76									
A. haemolyticus ATCC 19194	70	68	70	67	69	65	68	64	69	69	69	68	70	70	68	69	67	66	68	69	68	69	70	67	70	66	84										
A. sp. ATCC 27244	70	69	71	68	70	66	69	67	70	69	69	69	70	71	68	69	67	66	69	70	69	69	71	68	71	67											
A. calcoaceticus DSM 30006 (T)	66	70	78	76	77	74	78	66	77	76	76	77	77	80	77	77	76	74	77	83	78	78	79	81	88												
A. calcoaceticus RUH2202	70	74	83	80	81	78	83	69	82	81	81	82	82	85	83	83	81	79	82	87	82	81	85	87													
A. sp. DR1	68	74	84	79	80	76	82	66	80	82	82	82	81	84	83	81	82	79	82	88	80	80	82														
A. pittii SH024	70	72	84	81	81	79	84	69	84	82	82	82	84	84	82	83	81	78	82	87	86	88															
A. pittii DSM 9306	69	70	81	79	79	77	81	69	81	79	79	79	80	82	79	79	79	75	78	84	85																
A. pittii DSM 21653 (T)	69	71	81	78	79	76	80	66	80	79	79	79	79	82	78	79	78	76	79	86																	
A. calcoaceticus PHEA-2	71	76	84	81	84	80	85	68	84	83	82	84	83	88	84	84	83	80	84																		
A. baumannii W7282	69	71	82	80	83	81	85	72	84	85	85	86	86	87	90	90	93	93																			
A. baumannii W6976	67	69	79	78	79	77	81	70	80	81	81	83	82	83	86	86	89																				
A. baumannii TCDC-AB0715	68	70	82	79	82	80	85	70	83	87	87	87	85	86	92	91																					
A. baumannii 6014059	69	72	82	80	83	81	87	70	86	85	85	86	87	87	92																						
A. baumannii ACICU	69	72	84	79	83	80	86	68	84	85	85	87	85	87																							
A. baumannii AB307-0294	70	74	85	82	85	82	88	70	86	90	90	91	91																								
A. baumannii AYE	69	71	84	81	83	80	86	70	84	88	88	92																									
A. baumannii AB0057	68	72	83	80	83	81	86	70	84	89	89																										
A. baumannii 6013150	68	71	83	79	82	80	85	69	83	99																											
A. baumannii 6013113	68	71	83	79	82	80	85	69	83																												
A. baumannii AB900	69	71	83	81	82	82	86	70																													
A. baumannii SDF	65	67	69	68	70	68	69																														
A. baumannii ATCC 19606 (T)	69	72	83	81	84	82																															
A. nosocomialis NCTC 10304	66	68	78	78	80																																
A. baumannii ATCC 17978	70	73	81	79																																	
A. nosocomialis NCTC 8102	68	69	84																																		
A. nosocomialis RUH2624	71	72																																			
A. baylyi ADP1	74																																				

Additional File 6. Gene content comparison of the 38 strains in this study, table values represent the percentage of shared CDSs for each pair of strains. While strains from the same species generally share at least 80% of their CDSs, strains from different species can exhibit similar ratios (ratios ≥ 80% shown in gray). For example, *A. calcoaceticus* RUH2202 shares more than 80% of its CDS repertoire with DR1 and various *A. nosocomialis*, *A. baumannii*, *A. pittii* strains; PHEA-2 and DR1 share 88.1% of their CDSs, etc. Based on gene content only, *A. baumannii* SDF is distinct from all other *A. baumannii* strains in our study (shares at most 71.6%), which explains its placement in the K-string (Additional File 4) and genomic fluidity (Additional File 5) dendograms. These results indicate a potentially significant level of horizontal gene transfer among *Acinetobacter* species and illustrate an inability to delineate species based on gene content comparison only.
